# Association of Laparoscopic Methods and Clinical Outcomes of Cholecystolithiasis Plus Choledocholithiasis: A Cohort Study

**DOI:** 10.5152/tjg.2022.22110

**Published:** 2023-01-01

**Authors:** Hui Ji, Yafeng Hou, Xiaojian Cheng, Feng Zhu, Chao Wan, Lei Fang

**Affiliations:** 1 Department of Hepatological Surgery, Tongling People’s Hospital, Anhui, China

**Keywords:** Endoscopic retrograde cholangiopancreatography,, common bile duct primary suture,, laparoscopic cholecystectomy,, lithotomy of the common bile duct

## Abstract

**Background:**

Various surgical methods are available for cholecystolithiasis plus choledocholithiasis. The objective of this study is to explore the association between laparoscopic methods and clinical outcomes of cholecystolithiasis plus choledocholithiasis.

**Methods:**

This cohort study retrospectively included patients who underwent laparoscopic surgery for cholecystolithiasis plus choledocholithiasis at our hospital (January 2017 to March 2021). The primary outcome was bile leakage.

**Results:**

Totally 127 patients were enrolled. The time to get out of bed and the indwelling duration of the abdominal drainage tube in the patients who underwent laparoscopic cholecystectomy + lithotomy of common bile duct + common bile duct primary suture + endoscopic nasobiliary drainage were higher than the endoscopic retrograde cholangiopancreatography + laparoscopic cholecystectomy group, without differences in the laparoscopic common bile duct exploration group (all *P* < .05). All indexes decreased in the 3 groups after surgery (all *P* < .01). On the first day after surgery, only white blood cells (*P* < .001) and gamma-glutamyl transferase (*P* = .045) showed significant differences among the different surgical methods. The incidence of biliary leakage (*P* = .001) was higher in laparoscopic cholecystectomy + lithotomy of common bile duct + common bile duct primary suture + endoscopic nasobiliary drainage, while the occurrence of hyperamylasemia was higher with endoscopic retrograde cholangiopancreatography + laparoscopic cholecystectomy (*P* = .001). Compared with laparoscopic cholecystectomy + lithotomy of common bile duct + common bile duct primary suture + endoscopic nasobiliary drainage, laparoscopic common bile duct exploration was associated with fewer bile leakage (RR = 0.03, 95% CI: 0.003-0.37).

**Conclusion:**

Compared with laparoscopic cholecystectomy + lithotomy of common bile duct + common bile duct primary suture + endoscopic nasobiliary drainage, laparoscopic common bile duct exploration was associated with bile leakage.

**Main Points**

- There are many surgical methods to treat cholecystolithiasis and choledocholithiasis, all of which are safe and effective.
- Laparoscopic common bile duct exploration was negatively correlated with postoperative biliary leakage.
- The incidence of biliary leakage after laparoscopic cholecystectomy + lithotomy of common bile duct + common bile duct primary suture + endoscopic nasobiliary drainage is high, so it is necessary to improve the surgical techniques and control the surgical indications.

## Introduction

Cholecystolithiasis (gallstones) in Westernized countries are mostly cholesterol stones,^1^ forming due to an interplay of several factors, including underlying genetic predisposition, hepatic hypersecretion of cholesterol, alterations in metabolism, impaired gallbladder, gastrointestinal motility, and chronic inflammation.^2,3^ Risk factors for cholecystolithiasis include female gender, family history of gallstones, obesity, and rapid or cyclic weight loss.^1^ The reason for the formation of cholecystolithiasis may be associated with coronary artery disease, metabolic syndrome, and insulin resistance. Cholecystolithiasis formation mechanism is not completely clear but may be related to decreased gallbladder contraction and abnormal cholesterol metabolism. Most cholecystolithiasis are asymptomatic. A small number of patients with cholecystolithiasis (about 1.5% per year) are treated for cholecystolithiasis-related complications or symptoms.^4^ Cholecystolithiasis may cause severe epigastric or right upper quadrant pain, acute cholecystitis, acute cholangitis, or gallstone pancreatitis. Some small gallstones may be discharged into the common bile duct (CBD) through the cystic duct.^5,6^ Choledocholithiasis (gallstones in the CBD) may be asymptomatic but may lead to complications such as acute cholangitis or acute pancreatitis. Jaundice can occur if accompanied by biliary obstruction, and some patients without abdominal pain as the first symptom can also be treated due to yellow eyes and yellow urine.^7-10^ Choledocholithiasis is reported in approximately 4% of the general population and 10%-20% of patients undergoing cholecystectomy.^7,8^ The risk factors are for common bile duct stone formation cholecystolithiasis fell into the CBD, the infection of the intrahepatic bile duct stricture, and biliary ascaris. Considering the relatively lower prevalence of the 2 conditions, the occurrence of the 2 conditions is relatively rare; it is estimated that about 1%-15% of patients with cholecystolithiasis will also have choledocholithiasis.^11^

Cholecystectomy is advised for patients with symptomatic gallstones, and to prevent further progression of the disease, it is done within 7 days in mild acute cholecystitis, during early index admission for mild gallstone pancreatitis, and as early as possible (within 24 hours if possible) for biliary colic.^12-14^ Delayed cholecystectomy is recommended in severe acute cholecystitis or severe gallstone pancreatitis.^12-14^ Laparoscopic cholecystectomy is associated with a shorter hospital stay and period of convalescence than open cholecystectomy.^1,5,15^ Most gallstones form in the gallbladder and then migrate to the CBD as the gallbladder contracts. Stones may be discharged into the duodenal bowel cavity with bile flow or remain in the CBD. Therefore, the clinical manifestations of choledocholithiasis may be asymptomatic or associated with a variety of symptoms. The common methods for CBD stone clearance include endoscopic sphincterotomy following endoscopic retrograde cholangiopancreatography (ERCP) and laparoscopic bile duct exploration; in both procedures, CBD stone clearance is reported in >90% of the patients.^7-10^

At present, various treatment methods for cholecystolithiasis plus choledocholithiasis have been discussed.^8,16,17^ Among them, a classic surgical method is laparoscopic CBD exploration (LCBDTD), driven by the minimally invasive approach.^18^ Compared to ERCP and open surgery, LCBDTD has advantages like being safer, more reliable, earlier recovery, and more cost-effective.^19^ Endoscopic retrograde cholangiopancreatography can be used to remove CBD stones. During the same period (usually about 3 days after ERCP and without pancreatitis), laparoscopic cholecystectomy can be performed to reduce trauma in patients and avoid placing of a T-tube (endoscopic retrograde cholangiopancreatography + laparoscopic cholecystectomy [LCERCP]).^20^ Still, a nasal bile duct needs to be placed, and the surgery is performed in 2 sessions. Moreover, ERCP entails a risk of not removing the stones completely, especially in the case of large stones, and damages the sphincter of Oddi. Another surgical method is laparoscopic cholecystectomy + lithotomy of common bile duct + common bile duct primary suture + endoscopic nasobiliary drainage (LCBDPSENBD).^21^ Laparoscopic cholecystectomy + lithotomy of common bile duct + common bile duct primary suture + endoscopic nasobiliary drainage can solve all problems with 1 surgery: removing the gallbladder and the biliary stones, placing the nasal bile duct under direct vision of the choledochoscope, suturing the bile duct at one stage to avoid placing the T-tube, and reducing the irritation of the Oddis sphincter. Still, LCBDPSENBD has disadvantages such as bile duct inflammation or unskilled suture, and the incidence of bile leakage is relatively high. Therefore, the 3 surgical methods mentioned above are routinely performed clinically, and each has its advantages and disadvantages.

Hence, this study aimed to examine the clinical benefits, complications, and risk factors of LCBDTD, LCERCP, and LCBDPSENBD for patients with cholecystolithiasis plus choledocholithiasis.

## Material and Methods

### Study Design and Patients

This retrospective cohort study enrolled patients who underwent laparoscopic surgery for cholecystolithiasis plus choledocholithiasis at the Department of Hepatobiliary Surgery of Tongling People’s Hospital from January 2017 to March 2021.

The inclusion criteria for the study include patients who had (1) a diagnosis of cholecystolithiasis plus choledocholithiasis by preoperative imaging examination and postoperative pathology and (2) who underwent LCBDPSENBD or LCBDTD or LCERCP surgery. The patients who underwent laparotomy underwent other surgeries (such as liver resection, appendectomy, etc.) simultaneously or with incomplete data were excluded.

European Society of Gastrointestinal Endoscopy (ESGE) suggests offering stone extraction to all patients with CBD stones. The European Society of Gastrointestinal Endoscopy recommends a convergent ERCP for cholecystectomy in patients with CBD stones. Intraoperative ERCP can be performed during laparoscopic cholecystectomy as a first-stage treatment for cholecystocholithiasis or after the failure of preoperative endoscopic attempts to remove CBDS. Guidelines recommend laparoscopic cholecystectomy within 2 weeks of ERCP in patients with CBD stones to reduce the rate of outcome and the risk of biliary event recurrence. In patients undergoing laparoscopic cholecystectomy, choledochoscope exploration of the CBD is a safe and effective technique for CBD stone clearance.^22^ According to the guideline, we divided patients into 3 groups randomly.

This study was approved by the Ethics Committee of Tongling People’s Hospital (2021004). The requirement for patients’ informed consent was waived due to the retrospective nature of the study.

### Data Collection

Patients’ demographic data, disease data, and surgery-related information were collected through the hospital’s electronic chart system. Demographic data consisted of age, sex, body mass index, past medical history (hypertension, diabetes, cerebral infarction, tuberculosis, hepatitis B, etc.), and history of abdominal surgery. Surgery-related information included operation time, blood loss, intraoperative blood transfusion, etc.

The clinical characteristics were liver function before surgery and on the first and third days after surgery, including alanine aminotransferase, total bilirubin, albumin, alkaline phosphatase, γ-glutamyl transferase (γ-GT), postoperative complications, etc. In addition, postoperative follow-up information was collected, including postoperative complications, postoperative blood transfusion, postoperative admission to the intensive care unit, stone recurrence, hospital stay, and hospital expenses.

### Outcomes

The primary outcome was bile leakage. The secondary outcomes were other perioperative indicators, including liver function indicators, postoperative complications, and postoperative blood transfusion.

### Statistical Analysis

Data were analyzed using Statistical Package for the Social Sciences 22.0 (IBM, Armonk, NY, USA). The continuous variables were tested for normality. The data conforming to the normal distribution were presented as mean ± standard deviation and were compared using one-way analysis of variance with the least significant difference (LSD) post hoc test. The data that did not conform to the normal distribution were presented as median (range) and were compared using the Kruskal–Wallis *U* test. To adjust for potential confounders, age, sex, chief complaint, comorbidities, laboratory indicators, surgery duration, amount of bleeding, pain score, time to get out of bed after the operation, exhaust duration, and indwelling duration of abdominal drainage tube were adjusted using multivariate regression analysis. Two-sided *P* <.05 were considered statistically significant.

## Results

### Characteristics of the Patients

Among the 176 patients who were eligible for this study according to the inclusion criteria, 49 patients were excluded and 127 patients were included in the study (LCBDTD, n = 68; LCERCP, n = 28; LCBDPSENBD, n = 31). The baseline data of the patients are shown in Table 1. The proportion of males was smaller in the LCBDPSENBD group compared to the LCBDTD and LCERCP groups (*P* = .010), CBD diameter was smaller in the LCEDRCP group (*P* < .001), and stone size was smaller in the LCERCP group (*P* = .025). There were no significant differences in the other variables.

### Risk of Bile Leakage

After adjusting for potential confounders, the RR of bile leakage of LCBDTD compared to LCBDPSENBD was 0.03 (95% CI: 0.003-0.37, *P* = .005); the comparison of LCERCP and LCBDPSENBD was not statistically significant (RR = 0.45, 95% CI: 0.04-5.27, *P* = .522) (Table 2).

### Surgical Outcomes

The surgical outcomes of the patients are summarized in Table 3. There were significant differences among the 3 groups in terms of surgery duration (LCBDPSENBD had the longest duration; *P* < .001 vs LCBDTD and LCERCP), blood loss (LCBDPSENBD had the largest blood loss; *P* = .005 vs LCBDTD and LCERCP), time to get out of bed after surgery (longer with LCBDPSENBD; *P* = .045 vs LCERCP), indwelling duration of the abdominal drainage tube (longer with LCBDPSENBD and shorter with LCERCP; *P* < .001 vs LCBDTD), and hospitalization expenses (in increasing order: LCBDTD, LCBDPSENBD, and LCERCP; *P* < .001 among the 3 groups) among patients who underwent different surgical methods.

### Liver Function

The differences in liver function indexes between the 2 groups of patients and the differences before surgery, on the first day after surgery, and on the third day after surgery are shown in Table 4. All indexes decreased in the 3 groups after surgery (all *P* < .01) (Table 4). On the first day after surgery, only white blood cells (the highest with LCBDTD and the lowest with LCERCP, *P* < .001) and γ-GT (lower with LCBDPSENBD than with LCERCP, *P* = .048) showed significant differences among the different surgical methods.

### Complications

Table 5 presents the complications. The rate of hyperamylasemia was higher in LCERCP (17.9%, *P* < .001 vs LCBDPSENBD and LCBDTD). The rate of bile leakage was higher in LCBDPSENBD (29.0%) compared to LCBDTD (4.4%) and LCERCP (3.6%) (*P* = .001).

## Discussion

The results suggest that LCBDTD was associated with bile leakage. The liver function indexes of the 3 surgical methods were improved after the operation. Compared with the other two groups, the average CBD diameter of patients in the LCBDTD was larger, while the average stone diameter of patients in the LCERCP was smaller. The pain score was more important in LCBDPSENBD. Laparoscopic cholecystectomy + lithotomy of common bile duct + common bile duct primary suture + endoscopic nasobiliary drainage was associated with a longer time before getting out of bed. Endoscopic retrograde cholangiopancreatography + laparoscopic cholecystectomy showed a shorter indwelling duration of the abdominal drainage tube. The incidence of biliary leakage was higher in LCBDPSENBD, while the occurrence of hyperamylasemia was higher in LCERCP.

Of importance, this study does not advocate a specific surgical method or negate the others. The liver function indexes could be improved after these 3 operations. Therefore, the selection of the surgical method should be made based on the characteristics of the patients and the surgeon’s experience. The CBD diameter was larger in LCBDTD, while the stones were smaller in LCERCP. The results could help select the proper method for patients with cholecystolithiasis plus choledocholithiasis. Still, for patients with gallstones simultaneously in the gallbladder and the CBD, 2 essential points must be determined: (1) the most optimal surgical method for clearing the CBD and (2) the most optimal method for biliary drainage and decompression.^16,23,24^ Of course, the selected method should optimize the benefits and limit the possible complications as much as possible.

The 3 surgical methods have their advantages and disadvantages.^8,16,17^ It is widely and clinically accepted that the difference in the incidence of biliary leakage is mainly due to the different suture methods of the bile duct and the different ways of biliary decompression.^25,26^ Bile duct leakage can endanger the life of the patients.^25,26^ In LCBDTD, a T-tube is left in the CBD after suturing the bile duct,^18^ directly forming bile duct decompression and external bile drainage. The incidence of bile leakage is thus small due to the small suture tension in the CBD. In LCERCP, before surgery, the CBD stones have been removed by ERCP, and so only the gallbladder needs to be removed during the actual surgery, without opening the CBD.^20^ Bile leakage caused by the leakage of the stump of the cystic duct is clinically rare, so the risk of bile leakage is among the lowest. In LCBDPSENBD, although the bile duct is decompressed through external bile drainage through the nasal bile duct, the diameter of the nasobiliary duct is too small, and the drainage effect is not good. In addition, many patients cannot tolerate the nasobiliary duct through the nasal cavity, and some patients even pull out the nasobiliary duct by themselves. The section of the nasobiliary duct in the CBD is short, generally less than 10 cm, so the patient’s careless traction after the surgery can cause the nasobiliary duct to protrude out of the CBD and fall into the duodenal intestinal cavity, losing the drainage effect. In addition, some patients’ bile is thick, thus it is easy to cause blockage when passing through the nasal bile duct. All of the above factors cause the effect of nasobiliary drainage to be less than expected.^27-30^ Therefore, the bile pressure in the bile duct of LCBDPSENBD is the highest among the 3 surgical methods, and the tension at the bile duct suture of LCBDPSENBD is the highest, increasing the risk of bile leakage.

In order to reduce the bile leakage of LCBDPSENBD, the authors believe that strict control and correction are needed, and patients with bile duct stones need to meet the following conditions before surgery: (1) the CBD should be significantly dilated and larger than 1.5 cm in diameter. (2) There should be no combined intrahepatic bile duct stones or bile duct stenosis. (3) The patient’s total bilirubin level should be slightly elevated but less than 3 times the upper limit of normal. (4) There should be no history of acute cholangitis recently (2 weeks). (5) During surgery, the bile should be clear, there should be no turbid biliary sludge, and the CBD stones should be removed totally under choledochoscopy.

This study has limitations. The sample size was small since all patients were from a single center. In addition, the hospital was a tertiary center, thus leading to a selection bias since only the complicated cases were transferred from primary and secondary hospitals. The study was retrospective, limiting the data that could be analyzed. Prospective studies might help define the characteristics of the surgical treatment of cholecystolithiasis plus choledocholithiasis.

In conclusion, in patients with cholecystolithiasis plus choledocholithiasis, the incidence of biliary leakage was higher in LCBDPSENBD, while the occurrence of hyperamylasemia was higher with LCERCP. Laparoscopic common bile duct exploration was independently associated with bile leakage.

## Figures and Tables

**Table 1.** Patient Characteristics and Surgical Results

**Characteristics**	**LCBDTD** **(n = 68)**	**LCERCP** **(n = 28)**	**LCBDPSENBD** **(n = 31)**	***P***
**Age (years)**	59.3 ± 16.3	57.4 ± 14.0	57.2 ± 18.5	.786
**Sex (male), n (%)**	35 (51.5)	16 (57.1)	5 (16.1)^*, #^	**.010**
**BMI (kg/m** ^2^)	22.9 ± 2.7	22.3 ± 2.0	22.7 ± 2.5	.502
**Previous surgeries**	16 (23.5)	7 (25.0)	11 (35.5)	.462
**Common bile duct diameter**	1.3 ± 0.4	1.0 ± 0.5^*^	1.2 ± 0.2^#^	**<.001**
**Number of stones (<3), n (%)**	45 (66.2)	21 (75.0)	21 (67.7)	.695
**Size of stones (cm)**	0.8 ± 0.5	0.5 ± 0.2^*^	0.7 + 0.6	**.025**

^*^P < .05 vs LCBDTD; ^#^*P* < .05 vs LCERCP.

LCBDTD, laparoscopic common bile duct exploration; LCERCP, endoscopic retrograde cholangiopancreatography + laparoscopic cholecystectomy; LCBDPSENBD, laparoscopic cholecystectomy + lithotomy of common bile duct + common bile duct primary suture + endoscopic nasobiliary drainage; BMI, body mass index.

**Table 2.** Relative Risk for Bile Leakage

Variables	RR	95% CI	*P*
Types of surgery^#^			
LCBDTD	0.034	0.003-0.365	**.005**
LCERCP	0.447	0.038-5.266	.522
LCBDPSENBD	Reference	Reference	1.000

LCBDTD, laparoscopic common bile duct exploration; LCERCP, endoscopic retrograde cholangiopancreatography + laparoscopic cholecystectomy; LCBDPSENBD, laparoscopic cholecystectomy + lithotomy of common bile duct + common bile duct primary suture + endoscopic nasobiliary drainage.

**Table 3.** Comparison of the Recovery of Patients with Different Surgical Treatments

**Characteristics**	**LCBDTD (n = 68)**	**LCERCP (n = 28)**	**LCBDPSENBD (n = 31)**	***P***
Pain score	2.1 ± 1.1	1.6 ± 0.7	2.3 ± 1.4^#^	.065
Surgery duration (minutes)	126 ± 44	136 ± 44	192 ± 80^*,#^	**<.001**
Amount of bleeding (mL)	15 ± 9	12 ± 6	20 ± 14^*,#^	**.005**
Intraoperative blood transfusion, n (%)	0	0	0	**---**
Stone residue (%)	1 (1.5)	0	0	>.999
Stone recurrence (%)	2 (2.9)	3 (10.7)	0	.090
Time to get out of bed after surgery (days)	2.0 ± 0.8	1.7 ± 0.6	2.2 ± 0.7^#^	**.045**
Exhaust duration (days)	2.2 ± 0.7	1.9 ± 0.7	2.3 ± 1.0	.066
Indwelling duration of abdominal drainage tube (days)	5.1 ± 1.3	2.7 ± 1.4^*^	5.5 ± 2.8^#^	**<.001**
Hospitalization duration (days)	11.5 ± 2.9	13.5 ± 6.3^*^	12.4 ± 3.1	.070
Hospitalization expenses (yuan)	21 898 ± 6133	37 895 ± 4722^*^	25 175 ± 6413^*,#^	**<.001**

^*^P < .05 vs LCBDTD; ^#^*P* < .05 vs LCERCP.

LCBDTD, laparoscopic common bile duct exploration; LCERCP, endoscopic retrograde cholangiopancreatography + laparoscopic cholecystectomy; LCBDPSENBD, laparoscopic cholecystectomy + lithotomy of common bile duct + common bile duct primary suture + endoscopic nasobiliary drainage.

**Table 4.** Changes in Laboratory Indicators Before and After Different Surgeries

**Liver Function Index**	**LCBDTD (n = 68)**	**LCERCP (n = 28)**	**LCBDPSENBD (n = 31)**	***P***
**ALT (U/L)**				
Before surgery	177.09 ± 195.07	227.42 ± 225.89	221.74 ± 202.19	.427
The first day after surgery	114.25 ± 144.65	144.93 ± 148.03	121.84 ± 115.10	.617
The third day after surgery	73.96 ± 75.11	76.96 ± 70.15	67.19 ± 53.36	.814
*P*	<.001	.003	<.001	
**TB (mmol/L)**				
Before surgery	53.12 ± 61.09	55.20 ± 69.87	42.93 ± 43.36	.672
The first day after surgery	43.36 ± 52.39	54.50 ± 67.53	32.29 ± 29.14	.262
The third day after surgery	35.54 ± 41.89	34.66 ± 42.78	19.83 ± 11.48	.136
*P*	<.001	.001	.006	
**ALB (g/L)**				
Before surgery	39.60 ± 4.82	39.29 ± 5.97	40.81 ± 5.06	.457
The first day after surgery	35.72 ± 4.39	36.12 ± 5.81	35.96 ± 4.65	.925
The third day after surgery	36.83 ± 4.31	36.46 ± 4.01	37.45 ± 5.15	.685
*P*	<.001	.037	<.001	
**WBC (10 ^9^ /L)**				
Before surgery	5.99 ± 2.36	6.09 ± 2.36	6.54 ± 2.34	.550
The first day after surgery	12.62 ± 4.10	8.32 ± 3.03^*^	11.96 ± 4.25^#^	**<.001**
The third day after surgery	7.87 ± 2.35	9.00 ± 4.68	8.22 ± 2.71	.239
*P*	<.001	.002	<.001	
**γ-GT (U/L)**				
Before surgery	403.34 ± 435.78	550.43 ± 515.37	388.94 ± 389.01	.281
The first day after surgery	244.96 ± 267.18	396.46 ± 357.49	249.81 ± 225.50^#^	**.048**
The third day after surgery	200.23 ± 173.64	225.43 ± 181.45	171.94 ± 134.11	.468
*P*	<.001	<.001	.001	
**ALP (U/L)**				
Before surgery	225.63 ± 204.78	207.71 ± 133.96	228.94 ± 168.82	.886
The first day after surgery	167.58 ± 134.48	180.89 ± 116.24	155.77 ± 93.14	.732
The third day after surgery	156.53 ± 94.54	126.14 ± 76.63	146.64 ± 87.96	.329
*P*	<.001	<.001	<.001	

^*^P < .05 vs LCBDTD; ^#^*P* < .05 vs LCERCP.

LCBDTD, laparoscopic common bile duct exploration; LCERCP, endoscopic retrograde cholangiopancreatography + laparoscopic cholecystectomy; LCBDPSENBD, laparoscopic cholecystectomy + lithotomy of common bile duct + common bile duct primary suture + endoscopic nasobiliary drainage; ALT, alanine transaminase; TB, total bilirubin; ALB, albumin; WBC, white blood cells; γ-GT, γ-glutamyl transferase; ALP, alkaline phosphatase.

**Table 5.** Comparison of the Safety of Different Surgical Methods

**Characteristics**	**LCBDTD (n = 68)**	**LCERCP (n = 28)**	**LCBDPSENBD (n = 31)**	***P***
**Complications, n (%)**				
Hyperamylasemia	0	5 (17.9)	1 (3.2)	**.001**
Electrolyte disturbance	10 (14.7)	3 (10.7)	5 (16.1)	.440
Poor bilirubin descent	7 (10.3)	3 (10.7)	2 (6.5)	.848
Bile leakage	3 (4.4)	1 (3.6)	9 (29.0)^*,#^	**.001**
Postoperative bleeding	3 (4.4)	0	1 (3.2)	.809
Acute respiratory failure	0	0	1 (3.2)	.465
Pulmonary infection	2 (2.9)	1 (3.6)	1 (3.2)	1.000
Others (cerebral infarction, gastrointestinal bleeding, hypoproteinemia)	2 (2.9)	0	2 (6.5)	.532
Postoperative blood transfusion, n (%)	0	0	1 (3.2)	.465
Admission to ICU after surgery, n (%)	0	0	1 (3.2)	.465

^*^P < .05 compared with LCBDTD; ^#^*P* < .05 compared with LCERCP.LCBDTD, laparoscopic common bile duct exploration; LCERCP, endoscopic retrograde cholangiopancreatography + laparoscopic cholecystectomy; LCBDPSENBD, laparoscopic cholecystectomy + lithotomy of common bile duct + common bile duct primary suture + endoscopic nasobiliary drainage; ICU, intensive care unit.
